# Assessment of Variables Related to the Risk of Severe Adverse Events in Cutaneous Melanoma Patients Treated with Immune Checkpoint Inhibitors

**DOI:** 10.3390/cancers16020250

**Published:** 2024-01-05

**Authors:** Kremena Petrova Trichkova, Franziska Görtler, Line Bjørge, Cornelia Schuster

**Affiliations:** 1 Faculty of Medicine, University of Bergen, 5009 Bergen, Norway; kremena.trichkova@uib.no; 2 Department of Oncology and Medical Physics, Haukeland University Hospital, Haukelandsveien 22, 5021 Bergen, Norway; 3 Department of Clinical Science, Centre for Cancer Biomarkers CCBIO, University of Bergen, Jonas Lies vei 87, 5021 Bergen, Norway; line.bjorge@uib.no; 4 Department of Obstetrics and Gynecology, Haukeland University Hospital, 5021 Bergen, Norway

**Keywords:** melanoma, immune-related adverse events, ipilimumab, pembrolizumab, nivolumab, colitis, hepatitis, pneumonitis

## Abstract

**Simple Summary:**

Malignant melanoma is an aggressive cancer with a globally increasing disease rate. Historically, overall survival has been less than one year for advanced disease with distant metastases. The implementation of immune checkpoint inhibitors has remarkably transformed the therapeutic landscape, yielding 40% to 50% five-year survival rates. However, this improvement comes with a cost, as more than 50% of patients may experience severe side effects. Since there is a lack of validated markers predicting a high risk of severe immune therapy-related side effects, we conducted a systematic literature review to investigate the association between patient baseline characteristics and the risk of serious side effects from immune checkpoint blockade. The highest risk was identified among patients treated with ipilimumab. Yet, available data were insufficient to calculate patient-specific risk for adverse events. This work displays the need to report information about side effects more explicitly.

**Abstract:**

Malignant melanoma is a prevalent and aggressive cancer, with globally increasing incidences. While immune checkpoint inhibitors (ICIs) have prolonged the survival of patients with advanced melanoma over the last decade, this improvement comes with the risk of severe immune-related adverse events (irAEs). This systematic review investigates patient baseline characteristics (BCs) as predictive factors for developing severe gastrointestinal, hepatic, and pulmonary irAEs in patients treated with ipilimumab (anti-CTLA-4) and/or nivolumab/pembrolizumab (anti-PD-1). A systematic literature search was conducted in the Ovid databases MEDLINE and EMBASE on 22 April 2022, following the PRISMA guidelines. Out of 1694 articles, 13 were included in the final analysis. We analyzed BCs and the occurrence of severe colitis, hepatitis, and pneumonitis in 22 treatment arms and 3 treatment groups: anti-CTLA-4 (*n* = 2904), anti-PD-1 (*n* = 1301), or combination therapy (*n* = 822). However, missing data preclude a direct comparison of individual BCs and the association to specific irAEs between studies. Descriptive analysis did not identify any significant association between median age, gender distribution, or performance status and severe colitis, hepatitis, or pneumonitis for any of the three treatment groups. We call for greater transparency and standardization in the reporting of patient-specific irAEs.

## 1. Introduction

Malignant melanoma is the most aggressive form of cutaneous cancer, with 325.000 cases estimated worldwide in 2020. Although incidence rates are higher in the elderly population, melanoma is among the most frequently occurring cancers worldwide, particularly in younger age groups [1,2,3]. Despite the rising global incidence rate of melanoma, great improvement in the five-year relative survival has been reported over the last decade, especially in patients with more advanced disease [1,2].

This striking success can be attributed to the implementation of immune checkpoint inhibitors (ICIs) in the treatment of advanced diseases over a decade ago. Ipilimumab, a human monoclonal antibody against cytotoxic T-lymphocyte antigen-4 (CTLA-4), was the first drug to improve survival in patients with advanced melanoma in a randomized Phase 3 trial from 2010 [4].

Anti-PD-1 monotherapy is recommended as first-line therapy for locally advanced or metastatic melanoma, based on superior survival and safety data compared to ipilimumab [5,6]. The combination regimen of nivolumab and ipilimumab has translated into the best overall survival, with 52% of patients alive after five years compared to 44% for nivolumab monotherapy and 26% for ipilimumab monotherapy [7]. Nevertheless, the drawback is the high number of treatment-related Grade 3 and 4 adverse events reported in 59% of patients receiving both nivolumab and ipilimumab compared to 23% for nivolumab and 28% for ipilimumab monotherapies [7].

One of the major challenges of ICI therapy is the considerable risk of severe (CTCAE Grade 3–5) irAEs that can affect any organ of the body, regardless of treatment response and with varying onset times [8,9]. Both clinical trials and real-world studies report Grade 3-4 irAEs in up to 60% of melanoma patients treated with different ICI regimens, while only 0.3% to 1.3% of ICI-treated patients experience fatal irAEs (Grade 5) [5,7,9,10,11,12]. Severe irAEs can result in significantly prolonged hospitalization as well as reduced self-care activities of daily living and various levels of disability in individual patients [13,14,15,16,17].

The frequency and difficulty of managing organ-specific irAEs can vary greatly [9]. Gastrointestinal (GI) irAEs, such as diarrhea and colitis, are frequent causes of both severe and fatal irAEs in all types of ICIs and are particularly prone to complications if left untreated [9,10,18]. In fact, GI irAEs account for 70% of fatal events among patients treated with ipilimumab monotherapy and 37% of patients treated with a combination regimen [10]. The incidence of severe GI toxicities is the lowest with anti-PD-1 therapy (1.7%), followed by the CTLA-4 inhibitor (12%), and highest with the combination regimen (16%) [9,19,20,21].

Hepatitis is a frequent complication of both ICI combination and single-agent therapy, reported in 25–30% and 2–10% of patients, respectively [22]. Severe AST and ALT elevations are reported in 6–9% for the combination regimen and 1–2% for ipilimumab and nivolumab monotherapies [23]. Hepatitis is also a common fatal irAE, accounting for 22% of deaths in patients treated with the combination of nivolumab and ipilimumab or nivolumab monotherapy, and 16% of deaths in the ipilimumab monotherapy group [10]. Pneumonitis, although with relatively uncommon all-grade toxicity, is mostly seen in patients receiving ICI combination therapy (10%) [24]. This potentially serious irAE can be challenging to manage and is a frequent cause of ICI-induced death, contributing to 35% of fatalities within the PD-1 group of a large meta-analysis that reported a total of 333 drug-related fatal AEs among this patient group [10].

Current knowledge on the potential severe irAEs raises questions about their risk and illustrates the urgent need for risk prediction for individual cancer patients to facilitate shared decision making in clinical care. Limited data have been published on the associations between patient characteristics and the risk of irAEs for melanoma patients. Nevertheless, recent studies suggest potential correlations between gender, age, performance status, and the development of irAEs [14,25]. A significantly increased risk of severe irAEs is reported in women compared to men, particularly following anti-CTLA-4 therapy [26]. Furthermore, endocrinopathies occur slightly more frequently in women [14]. On the other hand, men have a higher risk of ICI pulmonary toxicity when treated with anti-PD-1 agents and a higher incidence of neurologic and vascular irAEs [14,27].

Some studies report similar toxicity rates of ICI therapy among young and old cancer patients [25,28,29]. Surprisingly, older patients were found to have lower occurrence of severe irAEs, lower ICI discontinuation rates, and fewer hospitalizations in other studies [30,31]. However, a higher treatment discontinuation rate is reported for patients aged 90 years or older [29]. Data from a recent retrospective study suggest that performance status is a more accurate predictor of ICI tolerance than chronological age. ECOG 2 or higher was recently identified as an independent risk factor for developing ICI-induced interstitial lung disease in patients with lung cancer [32]. The risk stratification of ICI treatment is further complicated by combining ICIs with other anticancer therapies, such as targeted therapy for BRAF-positive melanoma or relatlimab (anti-LAG-3) [3,33,34]. Previous publications focus mainly on the assessment of a single risk factor, such as gender or age, for developing irAEs, and the majority of these studies rely on retrospective data or single studies. As a novel approach, this work systematically reviews clinical trials involving exclusively cutaneous melanoma patients and investigates the association of patient baseline characteristics and the risk for developing severe organ-specific irAEs. Lastly, to the best of our knowledge, this review is the first to concentrate on the potentially lethal side effects of colitis, hepatitis, and pneumonitis.

## 2. Materials and Methods

### 2.1. Search Methods

This review was performed in accordance with the PRISMA (Preferred Reporting Items for Systematic Reviews and Meta-Analyses) guidelines and has not been registered [35].

The PICO framework was used to identify the key components of the systematic literature search. On 22 April 2022, the Ovid databases MEDLINE and EMBASE were systematically searched for studies in English that provide data on serious immune-related adverse events (irAEs) in patients with cutaneous melanoma treated with immune checkpoint inhibitors (ICIs) in clinical trials. The search was designed using a combination of keyword terms and controlled vocabulary, including MeSH and Emtree. The search strategy included constituted variations of the following terms: “Cutaneous melanoma”, “PD-1-inhibitor”, “CTLA-4-inhibitor”, “Nivolumab”, “Ipilimumab”, “Pembrolizumab”, “Adverse event”, “Hepatitis”, “Colitis”, and “Pneumonitis”. Each of the included variations contributed to additional results. A complete list of the search terms and their combinations is available in Appendix A.

### 2.2. Selection Criteria

The inclusion and exclusion criteria for study selection were developed in terms of the PICO framework.

### 2.3. Study Design

Only studies published in English were included in this work. The following study designs were required for inclusion: Phase 1/2, 2, 3, or 4; retrospective; observational; or cohort studies. Phase 1 studies, pooled analyses, case reports with fewer than 20 participants, pilot studies, editorials, summaries, and reviews were excluded. The included studies provide information on study protocols, results, and baseline characteristics, for each intervention group. Studies using registry data without excluding subjects enrolled in clinical trials were not eligible.

### 2.4. Participants

The inclusion criteria for the participants were as follows: non-acral cutaneous melanoma; unresectable or metastatic disease (Stage III or IV); adults aged 16 years or older; no serious preexisting (active) autoimmune disease; and no prior treatment with either anti-CTLA-4 or anti-PD-1/PD-L1. Studies were excluded if more than 10% of the melanoma cases were acral, ocular, mucosal, or occult. If the melanoma subtype was not specified, the study was included if the available ethnicity data indicated that 90% or more of the subjects had non-acral cutaneous melanoma, for instance, in patients of “white” or “Caucasian” ethnicity or a predominantly European and/or North American origin. Studies in which patients were treated with adjuvant ICIs were excluded. Exclusion was also granted for studies reporting only organ-specific metastases, such as those reporting only liver metastases. Studies that did not disclose any previous treatment or specify the type of prior systemic treatment were excluded, unless the patients were treated in a real-life setting in 2013 or earlier; in that case, ICI naivety was assumed, and the study was included.

### 2.5. Intervention

Studies focusing on the three following ICIs were included: nivolumab, pembrolizumab, and ipilimumab. Combination of the mentioned ICI therapies was allowed. Studies using a non-concurrent application of two different ICIs or a combination of ICIs with other treatments, such as BRAF inhibitors, chemotherapy, or vaccines, were excluded.

### 2.6. Outcome Measures

The inclusion criteria for outcome measures were as follows: immune-related adverse events of CTCAE Grade 3-5 were reported; the onset of irAEs was within 12 months from the last treatment dose; and at least one of the three following organ systems were affected by irAEs: gastrointestinal, hepatic, or pulmonary. Studies were excluded if the type of irAE was not defined, the CTCAE grade was not specified, irAEs of Grade 3-5 were not reported, the toxicity status was not reported separately for the different intervention groups, or the toxicity status/assessment was available for fewer patients than described in the baseline characteristics.

### 2.7. Study Selection

All identified studies were independently screened by two reviewers (K.T. and C.S.), using the already established eligibility criteria. Firstly, the authors screened the titles and abstracts, and secondly, they reviewed the full text of all remaining publications. Any disagreement or uncertainty was resolved through discussion, and consensus was reached with a third coauthor (L.B.).

### 2.8. Definitions

The American Society of Clinical Oncology (ASCO) defines an irAE as any side effect associated with ICI treatment likely to be caused by its intended mechanism of action, which is immune system activation [22]. The publications included in this systematic review use either the term “treatment-related adverse events” or “immune-related adverse events” (irAEs) to describe the adverse immunological events following ICI therapy. In this study, the latter term was used.

The severity of irAEs in all included studies is graded according to the Common Terminology Criteria for Adverse Events (CTCAEs) and each included article refers to the relevant CTCAE version. Severe irAEs are defined as CTCAE Grade 3–5, where Grade 5 is a fatal condition [17].

According to the eighth edition of the *American Joint Committee of Cancer (AJCC) Staging Manual*, advanced or inoperable melanoma is defined as Stage III locoregionally advanced disease or Stage IV metastatic disease, and metastatic melanoma (Stage IV) is further categorized into four different M stages [36]. Metastases to distant lymph nodes, skin, or muscle are classified as Stage M1a. Stage M1b includes distant metastases to the lung, whereas any other visceral metastasis is categorized as M1c. Stage M1d involves CNS metastases [36]. We redefined Stage M1d based on the reported number of brain metastases in studies using an AJCC version before the eighth and latest edition [36].

### 2.9. Data Collection

For each intervention group, the following baseline characteristics were extracted: the number of participants, age (median and range), sex (male and female), the ECOG scale (Grade 0, 1, and 2+), BRAF status (pos/neg), PD-L1 status, and the M stage.

The incidences of CTCAE Grade 3–4 and 5 irAEs were collected for each intervention group for three organ systems, namely gastrointestinal, hepatic, and pulmonary, as our work focused on these three irAEs. As colitis and diarrhea are two gastrointestinal irAEs with similar and overlapping definitions, data on both were collected and merged [17]. Hepatitis of non-viral etiology is described by various CTCAE terms in the included articles, some describing the biochemical changes, others the etiology, and some the clinical result of hepatitis. Hence, data on the following hepatic irAEs were collected and merged: elevated AST or ALT, hepatitis, autoimmune hepatitis, toxic hepatitis, hepatotoxicity, acute hepatitis, and acute liver failure.

### 2.10. Data Analysis

We had planned to perform a meta-analysis and systematic review. Baseline characteristics and incidences of organ-specific irAEs of Grade 3 or higher were collected for each treatment arm. However, the published data did not provide BCs related to specific side effects. Attempts to obtain more detailed information from the authors of the included studies remained unsuccessful. Therefore, the statistical analyses of this paper are mainly descriptive and indicative. Study heterogeneity and estimated common effects are visualized in forest plots. The analyses were conducted using the metafor package in R 4.3.2 [37]. The Clopper–Pearson interval was used to calculate the 95% confidence intervals for the individual studies [38]. To estimate the between-study variance, we used the DerSimonian–Laird random-effect model [39].

## 3. Results

### 3.1. Search Results and Study Inclusion

**Figure 1:**
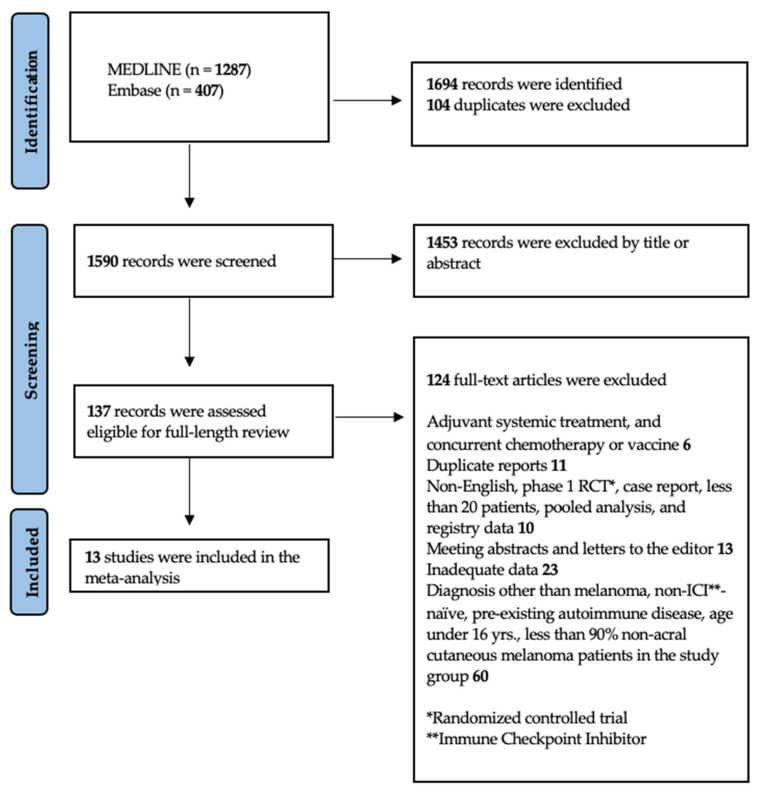
PRISMA flow diagram of study inclusion [35].

The systematic literature search was conducted following the PRISMA guidelines; detailed information is provided in the PRISMA flow diagram in Figure 1. The search includes articles published from 2003 to 22 April 2022, which yielded a total of 1694 articles. After deduplication in EndNote (title, author, publication year, and subtitle), 1590 articles remained. Following the primary screening of titles and/or abstracts and further removal of duplicates, 137 articles were selected for secondary screening. Eighteen publications met all the selection criteria, before excluding five additional trial duplications. One article [40] was excluded as the real-life patient cohort was also used in another selected article [41]. Three articles [5,42,43] provided analyses of data from the KEYNOTE-006 study. The 2019 publication was included, due to reporting the most detailed account of irAEs. A fourth article [44] was excluded as the population was reanalyzed in a subsequent publication of the Checkmate-067 trial [45], which reported substantially more instances of irAEs still within the 12-month window set in the selection criteria. Finally, the study by Hodi et al. [46] was excluded as irAEs only were reported for the first 30 days after the last treatment dose, compared to the 100-day safety window of its foregoing publications [47].

In total, 13 studies published between 2010 and 2021 were included after the final full-text review. Both retrospective studies and randomized trials were included. One study reported data from an expanded access program in Germany [48].

### 3.2. Description of Included Studies

The included studies and interventions are listed in Table 1.

### 3.3. Descriptive Analyses of Baseline Characteristics and Immune-Related Adverse Events

To conduct descriptive analyses of baseline characteristics and immune-related adverse events, we pooled all patients and categorized them into three groups based on treatment with CTLA-4 inhibition (*n* = 2904), PD-1 inhibition (*n* = 1301), or combination therapy (*n* = 822). Among the 13 included studies, we identified 22 treatment arms. Nine studies reported ipilimumab monotherapy; four studies described nivolumab or pembrolizumab monotherapy; and in four studies, combination therapy was applied.

#### 3.3.1. Grade 3-4 Immune-Related Adverse Events

**Figure 2:**
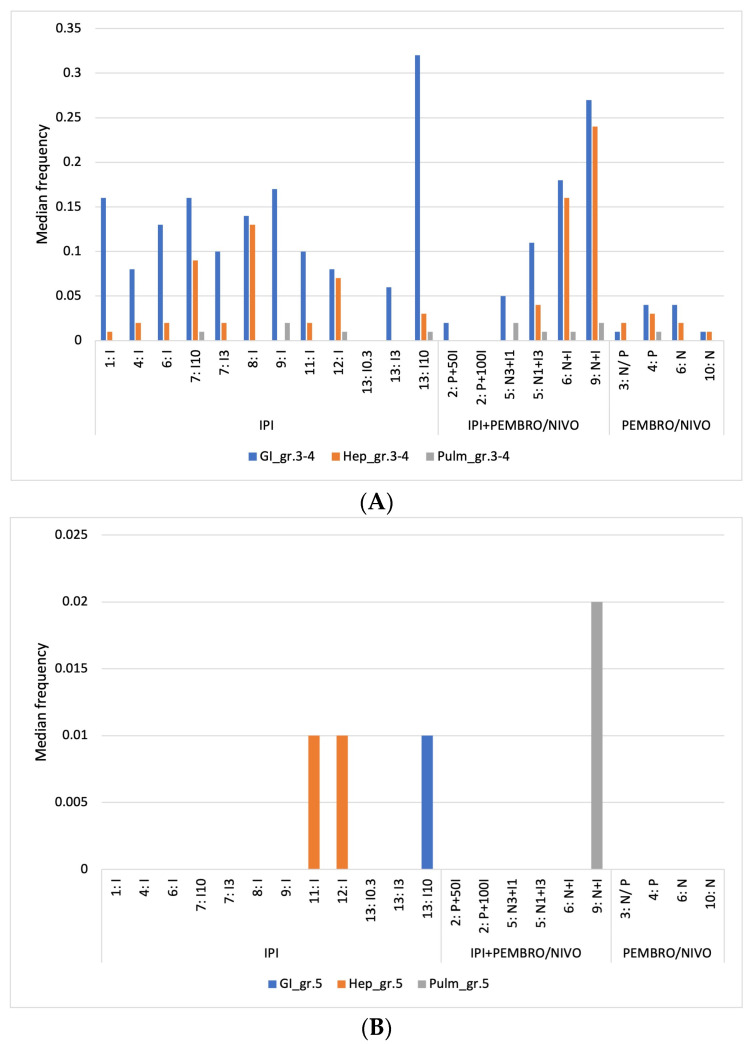
Severe immune-related adverse events by treatment arm: (**A**) Grade 3–4; (**B**) Grade 5.

Figure 2A visualizes the gastrointestinal (GI), hepatic (Hep), and pulmonary (Pulm) AEs of CTCAE Grade 3-4 for each treatment arm. Gastrointestinal AEs were dominant in every treatment group, while pulmonary AEs accounted for the lowest incidence. The grouped median frequency of hepatic and pulmonary AEs was similar across the three different treatment groups: 2% (min. 0%, max. 24%) for hepatitis and 0% to 1% (min. 0%, max. 2%) for pneumonitis. In comparison, GI AEs had a higher grouped median of frequency. The highest frequency of GI irAEs was observed for IPI monotherapy (12%), followed by 8% for the combined treatment and 3% for the PD-1 group. There was little difference in the frequency of hepatitis (2%), pneumonitis (0%), and colitis (1%) for the IPI monotherapy when the three study arms with IPI 10 mg were excluded. The total incidence of Grade 3–4 AEs was higher for the IPI monotherapy and combination therapy groups compared to the PD-1 group. Figure 2A illustrates the median frequencies of Grade 3–4 events for the three subgroups. The groups’ median frequencies were 16% for the IPI monotherapy group, 12% for the combination therapy group, and 5% for the PD-1 group.

**Figure 3:**
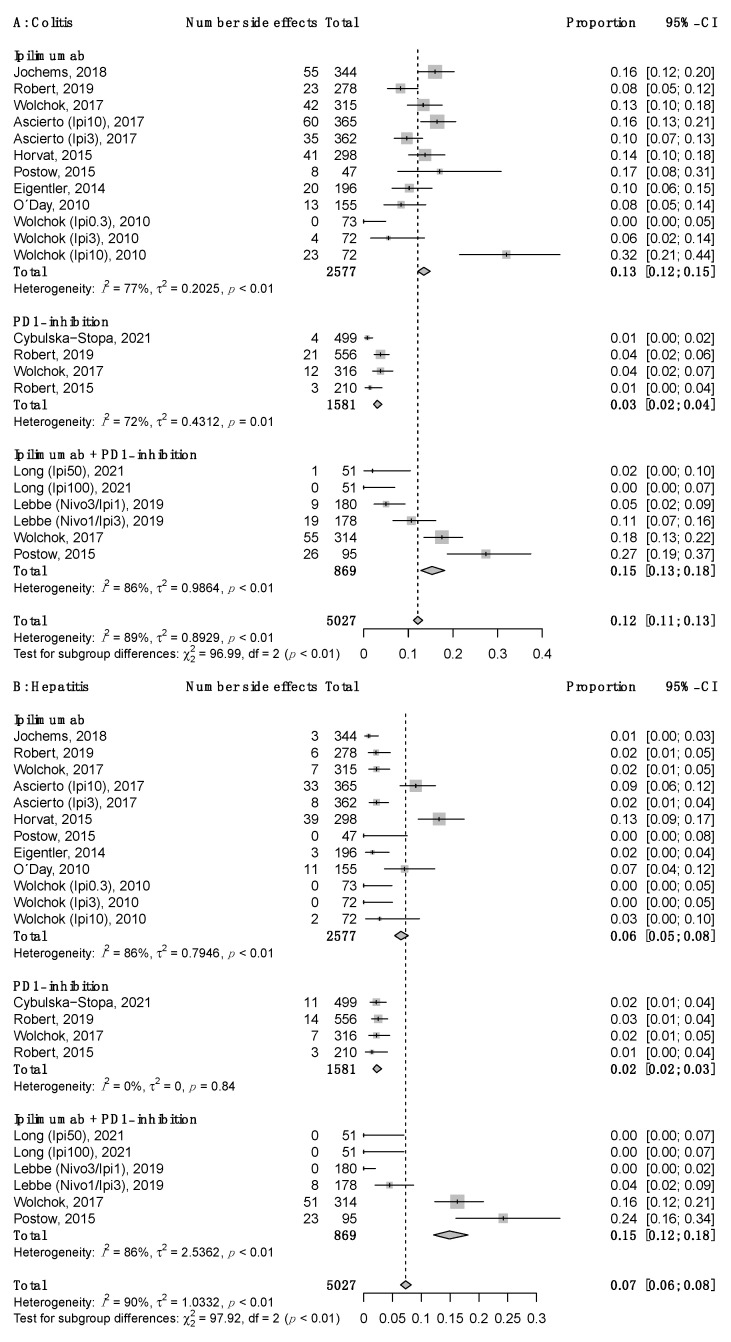

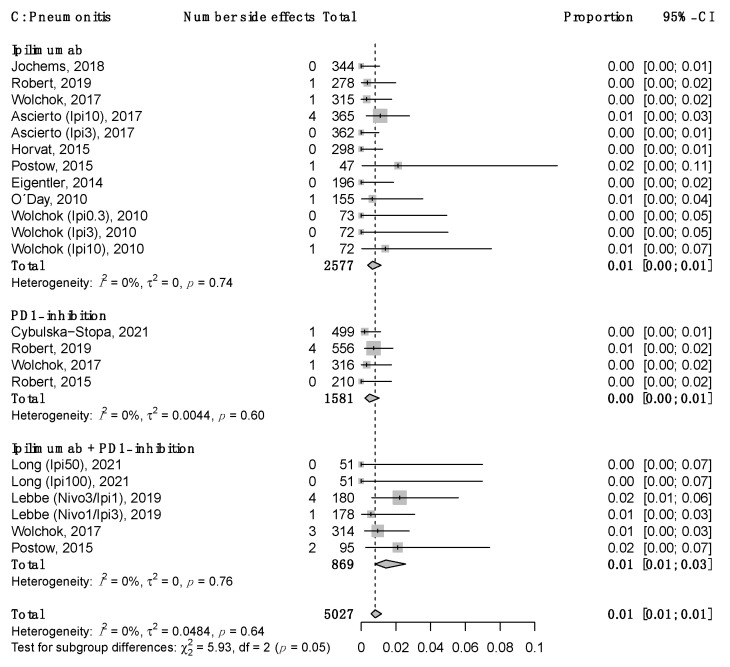
Grade 3-4 immune-related adverse events are shown for all studies included in the three treatment groups: ipilimumab, PD-1 inhibition, and ipilimumab + PD-1 inhibition. Based on the treatment group, the side effects of colitis (**A**), hepatitis (**B**), and pneumonitis (**C**) are visualized with the corresponding confidence interval in a forest plot [11,41,42,45,47,48,49,50,51,52,53,54,55].

Figure 3A–C illustrate the estimated common effects by organ-specific Grade 3–4 irAEs and interstudy heterogeneity. The estimated common proportion of Grade 3–4 organ-specific irAEs was highest for colitis. We observed similar results for the ipilimumab monotherapy and combination regimen (13% vs. 15%, respectively) and a much lower side effect rate of 3% for PD-1 monotherapy. The results are shown in Figure 3A. For hepatitis, the estimated total proportion of Grade 3–4 events significantly varied with the type of agent, namely 6% for ipilimumab, 15% for the combination regimen, and 2% for nivolumab/pembrolizumab, as visualized in Figure 3B. Grade 3-4 pneumonitis was estimated to occur equally rarely across all three treatment groups, with 1% reported in the IPI monotherapy and combination groups and 0% in the PD-1 group, as shown in Figure 3C. Pneumonitis had a low interstudy heterogeneity in all three treatment groups, visualized by overlapping 95% confidence intervals and supported by an I^2^ value of 0% and non-significant *p* values for heterogeneity (Figure 3C). The interstudy heterogeneity for colitis and hepatitis was significant for five of the six subgroups, with I^2^ values ranging from 72% to 86% (Figure 3A,B). Based on visualized data, studies no. 13 [55], no. 6 [45], and no. 9 [47] were considered outliers, as their true proportions stood out among their respective subgroups’ common proportion, as shown in Figure 3A,B. Like study no. 13 [55], studies no. 7 [52] and no. 12 [54] also employed the more toxic regimen (10 mg IPI), but they did not deviate from the estimated common proportion. As is clearly visible in Figure 3A–C, PD-1 inhibition caused the lowest incidence of G3-4 irAEs among the three treatment groups.

#### 3.3.2. Grade 5 Immune-Related Adverse Events

Figure 2B shows the frequency of CTCAE Grade 5 adverse events. Some studies have frequencies lower than 1% due to low incidences and are therefore not captured in the bar chart. Only four study arms reported Grade 5 AEs equal to or higher than 1% of the patient population. Three of these events were registered in the IPI group and one in the combination group. Two out of these four reports described hepatic Grade 5 AEs: one was a gastrointestinal event and the other a pulmonary event.

#### 3.3.3. Age

**Figure 4:**
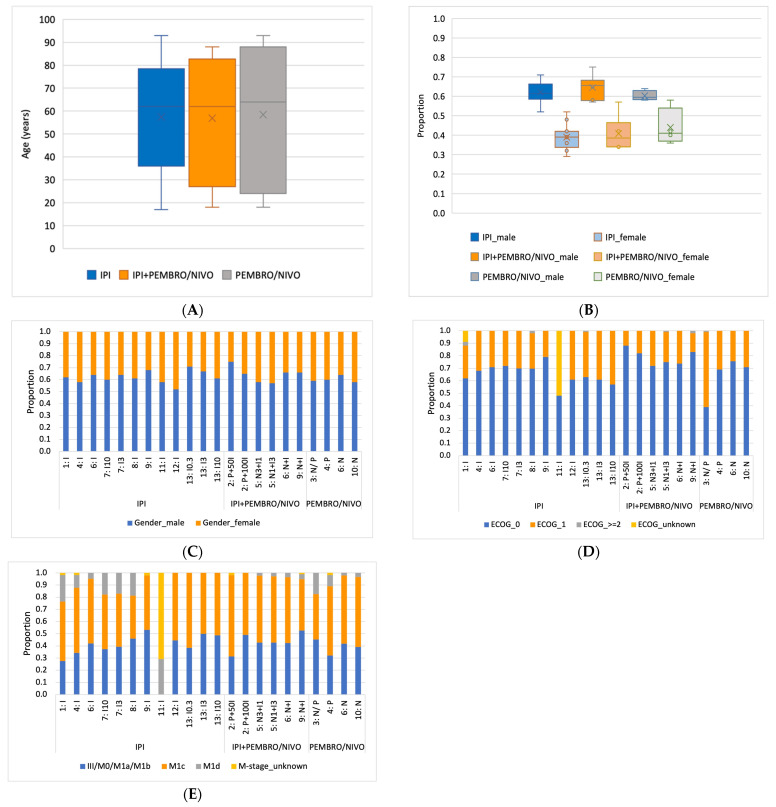
Descriptive data, distribution by treatment group in box plots and by treatment arm in stacked charts: (**A**) age; (**B**) gender distribution for each of the three treatment groups visualized in box plot; (**C**) gender distribution for each single treatment arm; (**D**) ECOG status; (**E**) M stage.

Figure 4A illustrates box plots summarizing the median age, as well as the minimum and maximum values, for each of the three treatment groups. Study no. 4 [42] was excluded from the analysis due to reporting the interquartile range and missing minimum/maximum ages. The median age for the ipilimumab monotherapy group was 62 years (min. 17–max. 93 years); for the PD-1 monotherapy group, it was 62 years (min. 18–max. 88 years); and for the combination group, it was 64 years (min. 18–max. 93 years). Based on the provided confidence intervals, it is evident that the ages of the youngest and oldest patients are similar for the three treatment groups. However, the IPI monotherapy subgroup is distinctive in having fewer patients in the highest and lowest age groups, as visualized by a narrower box plot. The real-world studies no. 3 [41] and no. 8 [53] reported the highest maximum age of 93 years. The maximum age reported in the two other real-world studies, study no. 1 [49]) and study no. 11 [48], is 79 years and 85 years, respectively, which is close to the maximum age reported by the randomized controlled trials within the IPI group.

#### 3.3.4. Gender

Within the IPI group, 62% of participants were male, and 38% were female. Among the patients treated with combination therapy, 66% were male. In one arm of study no. 2 [50], 75% of the enrolled individuals were male, which represents the highest percentage of male subjects registered within the three treatment groups. Finally, the PD-1 treatment group had the lowest overall male participation, with 60% male and 40% female patients, as visualized in Figure 4B. The stacked bar chart in Figure 4C shows the dominant proportion belonging to male participants, accounting for more than 50% of patients in each of the 22 study arms.

#### 3.3.5. ECOG Status

Figure 4D presents patients’ ECOG status, which was grouped into four categories based on the differentiation in the included studies: ECOG 0, 1, 2, or higher, or unknown. Since study no. 11 [48] placed patients with ECOG 1 and 2 in the same category, these patients were included in the category with unknown ECOG status in our analysis. In addition, only study no. 1 [49] involved patients with unknown ECOG status. Except for study no. 3 [41] in the PD-1 group and study no. 11 [48] in the IPI group, most patients were ECOG 0. This applied to 66% of patients in the IPI group, 70% in the combination group, and 79% of patients in the PD-1 group. ECOG 2 or higher was only present in a few treatment arms (studies no. 1, 3, 5, 8, 9, and 13) and constituted a small amount of the patient population. The real-world studies no. 3 [41] and no. 11 [48] reported a considerably lower ECOG 0 frequency than the pooled treatment groups’ median frequency within the IPI and PD-1 groups. In contrast, the ECOG 0 frequency of the two other real-world studies, no. 1 [49] and no. 8 [53], was close to the median of the pooled IPI group, with 62% and 69%, respectively.

#### 3.3.6. Classification of Metastasis

The presented results are based on the AJCC8 classification of the M stage, as described in Section 2.8 [36]. Figure 4E illustrates the distribution of the M stage by treatment arm. Across all three treatment groups, most patients had Stage M1c disease: 49% in the IPI group, 54% in the combination group, and 57% in the PD-1 group. Stage III-M1b was reported for 41% of patients in the IPI group, 43% in the combination group, and 40% in the PD-1 group, as shown in Figure 4E. The frequency of CNS metastases (M1d) was similarly low for all three groups, with 8% (IPI), 3% (combination), and 6% (PD-1), respectively. An unknown M stage was reported in a few study arms and for under 5% of the patients in each treatment group, except for the real-world study no. 11 [48], which reported an unknown M stage in 71% of patients.

#### 3.3.7. BRAF and PD-L1 Status

**Figure A1:**
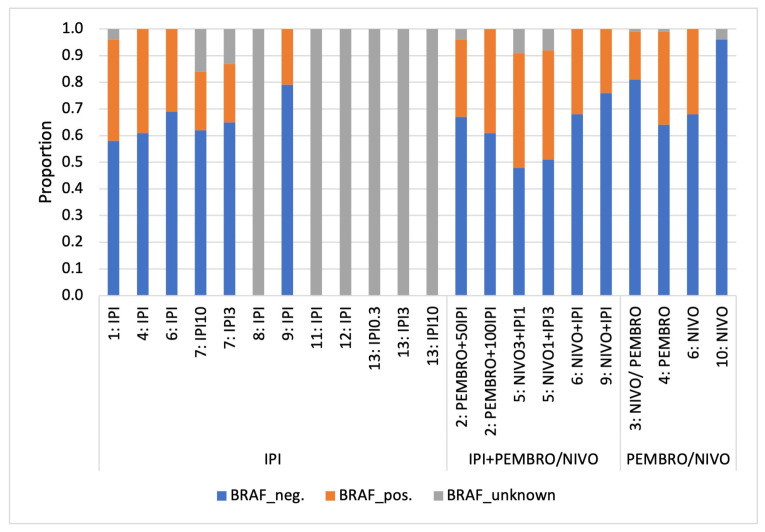
Distribution of BRAF status by treatment arm.

Figure A1 in Appendix B shows that six of the twelve IPI study arms lack information about BRAF mutation status. Additionally, an unknown BRAF status was reported in most studies in the PD-1 inhibition and combination therapy groups. Among the studies reporting BRAF status, at least 50% of patients had a BRAF wild-type status. Due to missing data, BRAF status frequencies could not be compared across the three treatment groups.

**Figure A2:**
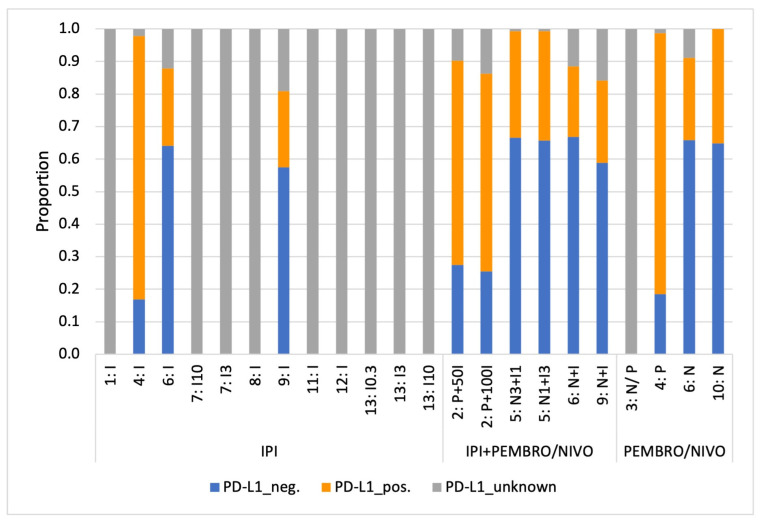
Distribution of PD-L1 status by treatment arm.

Data regarding PD-L1 status were even more sparse, with missing information in 10/22 included study arms, as shown in Figure A2 in Appendix B. An unknown PD-L1 status was reported in the majority of IPI treatment arms. In addition, the PD-L1 status was quite heterogeneous among the reported treatment arms for patients receiving either combination or PD-1 treatment. Due to a lack of data, the different treatment arms could not be compared.

## 4. Discussion

In this systematic review, we investigated the association between baseline characteristics and severe (Grade 3–5) organ-specific irAEs, namely colitis, hepatitis, and pneumonitis. Our aim was to assess the variables related to the risk of severe AEs in cutaneous melanoma patients treated with immune checkpoint inhibitors. Detailed information connecting baseline patient characteristics to specific irAEs could not be obtained for the included studies. To enhance our knowledge of the causes of irAEs, we encourage researchers to provide more explicit reporting of severe AEs based on patient characteristics.

In our analysis focusing on colitis, hepatitis, and pneumonitis, 5.1% of patients treated with PD-1 inhibition had G3-4 irAEs compared to 17.3% in the IPI monotherapy group and 23.2% in the combination regimen group. A low incidence of severe irAEs for PD-1 inhibition is consistent with previously published findings. In general, the incidence of G3-4 irAEs is slightly lower in our study material than in other publications [9,10,11,21,24]. This difference may be due to different selection criteria in each meta-analysis or study. In addition, single studies using more toxic regimes will report a higher incidence of severe irAEs. When considering each of the analyzed side effects, colitis was the most common G3-4 irAEs across all three treatment groups in our analysis. This is in line with previous Phase 3 trials showing that colitis is the most frequently occurring irAE and that the combination of ipilimumab and nivolumab has the highest incidence of both total and severe gastrointestinal adverse events [9,10,21]. However, our data revealed the highest median frequency of severe colitis among the IPI monotherapy group, with 12% of patients experiencing GI irAE (G3-4) compared to 8% in the combination group and 3% in the PD-1 group. On the other hand, the estimated common proportion of G3-4 colitis was 15% for the combination regimen group compared to 13% for the ipilimumab monotherapy group. Similarly, severe hepatic and pulmonary irAEs have been reported to occur more frequently in patients treated with the combination regimen [22,23,24]. Grade 3–4 AST and ALT elevation were reported in 6–9% for the combination regimen and 1–2% for ipilimumab and nivolumab monotherapies [23]. A retrospective study identified Grade 3-4 pneumonitis in 3% of patients treated with the combination of ipilimumab and nivolumab compared with 0.7% in the anti-PD-1/PD-L1 group [24]. A systematic review focusing on PD-1-related pneumonitis reported 0–4.1% G3-5 events for combination therapy in melanoma studies [56]. Contrary to other reports, our data show similar median frequencies of Grade 3–4 irAEs across the three treatment arms for both hepatitis and pneumonitis: 2% (min. 0%, max. 24%) and 0 to 1% (min. 0%, max. 2%). However, the estimated common proportion of Grade 3–4 hepatitis was higher for both ipilimumab monotherapy and the combination of ipilimumab and nivolumab, at 6% and 7%, respectively. The proportion of Grade 3–4 pneumonitis was comparable to the median frequency, estimated to be 0–1%. Fatal (Grade 5) irAEs occurred in 0–2% of patients across the three ICI treatment arms in our analysis, which is close to 0.3–1.3% of Grade 5 irAEs in the Vigilyze database [10].

Furthermore, we observed higher frequencies of fatal (Grade 5) irAEs in the IPI monotherapy and combination groups compared to the PD-1 group. Data from the international database Vigilyze show that colitis, hepatitis, and pneumonitis are among the most common causes of ICI-associated death for ipilimumab, anti-PD-1, and ipilimumab–nivolumab. Hepatitis was reported to be a frequent cause of immune-related death in the PD-1 monotherapy and IPI combination therapy groups, accounting for 22% of deaths for each of them [10]. Colitis was reported as the dominant cause of fatal toxic events for the IPI monotherapy group (70%), and pneumonitis was observed as the most common fatal irAE for the PD-1 treatment group (35%) [10]. Among the included studies in our analysis, Grade 5 AEs were reported in a small number of studies. Only four treatment arms reported Grade 5 events equal to or higher than 1% of the study arm population. Death was reported for ≥1% within the IPI monotherapy and combination groups. This is consistent with previous reports of higher incidences of severe colitis, hepatitis, and pneumonitis for these treatment groups compared with PD-1 monotherapy [9,22,24]. In contrast, among the studies included in this review, both hepatic Grade 5 irAEs were reported in the IPI monotherapy group. Thus, in our review, hepatitis was the dominating cause of ipilimumab-associated death, not colitis. Furthermore, no Grade 5 colitis was registered in the combination group. In conclusion, despite expectations, we identified the highest incidence of colitis and the highest incidence of irAE-related death among patients treated with ipilimumab monotherapy.

The baseline characteristics of age, sex, and ECOG status were comparable across the included studies. Yet, their impact on the occurrence of irAEs could not be calculated due to a lack of detailed information. Interestingly, male participants had predominance in all three treatment groups, with the highest rate in the combination regimen. Since all studies included in the combination group were randomized trials, this raises speculation if male candidates were more often considered suitable for potentially more toxic treatment options compared to females. Most participants in the included studies had ECOG 0, apart from the two real-world studies, no. 3 [41] and no. 11 [48]. Since the latter was based on an expanded access program for ipilimumab, this might explain the higher percentage of patients with ECOG 1-2 included due to the lack of other promising treatment options at this time. Based on the available data, the correlation of the M stage, BRAF mutation status, and PD-L1 expression to severe irAEs could not be analyzed. In particular, the reporting of the latter two was incomplete across the included studies. This is probably due to a lack of standard testing before BRAF/MEK inhibition became approved, and the fact that PD-1 inhibition is given to patients independently of its expression in tumor tissue [4,45,57]. Future investigations may evaluate the possible predictive value of BRAF and PD-L1 expression for determining the risk of irAEs.

There are multiple limitations to this systematic review. While the data obtained for the analyses were collected through a systematic study selection, missing patient-specific data precluded a direct comparison of individual baseline characteristics and the association to specific irAEs between the different studies. Furthermore, calculations of uncertainty or correction for population composition could not be performed based on the available data. The heterogeneous reporting of irAEs among the studies resulted in the exclusion of several studies that otherwise would be eligible. Not all studies reported organ-specific severe irAEs separately from total irAEs and were therefore not qualified. Finally, the number of studies per treatment group varied. Ipilimumab-containing treatment was represented to a higher extent than PD-1 treatment. This reflects the higher number of studies including ipilimumab within the defined study period. On the other hand, a strength of this systematic review is the use of well-defined selection criteria to ensure a homogenous study population in the included trials. Predefined variables from tables of BCs were chosen since most studies reported a relatively standardized set of patient characteristics. Additionally, our meta-analysis excluded all studies that only included patients with one type of distant metastasis, such as solely patients with liver metastases. Altogether, these choices ensured a homogenous patient population across studies and treatment groups.

## 5. Conclusions

In conclusion, our review did not reveal any clear associations between predefined patient BCs and the risk of developing severe organ-specific irAEs like colitis, hepatitis, and pneumonitis in patients treated with ICIs. The current reporting of BCs and irAEs is insufficient to perform analyses and risk stratification since the association of these two variables cannot be investigated on a patient-specific level. We suggest publishing the occurrence of severe irAEs in more detail. This might include a table containing the number of ≥G3 irAEs within ECOG status and age groups as well as gender-specific information. The addition of M stage or other BCs might also be considered. In essence, our work underscores the importance of transparent and standardized reporting of patient-specific irAEs to develop reliable risk stratification tools in clinical practice. This will contribute to supporting shared decision making, optimizing personalized treatment, and ensuring the effective use of healthcare resources in times of continuously increasing numbers of treatable patients.

## Figures and Tables

**Table 1:** Included studies and interventions.

Article No., Year	Title	Study	Author	Treatments Arms (ICI)	No. of Patients
1, 2018	Real-world use, safety, and survival of ipilimumab in metastatic cutaneous melanoma in the Netherlands	Retrospective, The Netherlands	Jochems, A. [49]	IPI	344
2, 2021	Standard-Dose Pembrolizumab Plus Alternate-Dose Ipilimumab in Advanced Melanoma: KEYNOTE-029 Cohort 1C, a Phase 2 Randomized Study of Two Dosing Schedules	NCT02089685 KEYNOTE 029 (Cohort 1C)	Long, V. [50]	PEMBRO + IPI Two dose schedules	51/51
3, 2021	First-line treatment of advanced/metastatic melanoma with anti-PD-1 antibodies: multicenter experience in Poland	Retrospective, Poland	Cybulska-Stopa, B. [41]	NIVO/PEMBRO	308/191
4, 2019	Pembrolizumab versus ipilimumab in advanced melanoma (KEYNOTE-006): post hoc 5-year results from an open-label, multicentre, randomised, controlled, phase 3 study	NCT01866319 KEYNOTE 006	Robert, C. [42]	IPI/PEMBRO	556/278
5, 2019	Evaluation of Two Dosing Regimens for Nivolumab in Combination with Ipilimumab in Patients with Advanced Melanoma: Results From the Phase IIIb/IV CheckMate 511 Trial	NCT02714218 CheckMate 511	Lebbe, C. [51]	NIVO + IPI Two dose schedules	180/178
6, 2017	Overall Survival with Combined Nivolumab and Ipilimumab in Advanced Melanoma	NCT01844505 CheckMate 067	Wolchok, J.D. [45]	NIVO/IPI/NIVO + IPI	314/316/315
7, 2017	Ipilimumab 10 mg/kg versus ipilimumab 3 mg/kg in patients with unresectable or metastatic melanoma: a randomised, double-blind, multicentre, phase 3 trial	NCT01515189	Ascierto, P.A. [52]	IPI Two dose schedules	365/362
8, 2015	Ipilimumab 10 mg/kg versus ipilimumab 3 mg/kg in patients with unresectable or metastatic melanoma: a randomised, double-blind, multicentre, phase 3 trial	Retrospective, USA	Horvat, Z. [53]	IPI	298
9, 2015	Nivolumab and ipilimumab versus ipilimumab in untreated melanoma	NCT01927419	Postow, M.A. [47]	IPI/NIVO + IPI	95/47
10, 2015	Nivolumab in previously untreated melanoma without BRAF mutation	NCT01721772 CheckMate 066	Robert, C. [11]	NIVO	210
11, 2014	Effectiveness and tolerability of ipilimumab: experiences from 198 patients included in a named-patient program in various daily-practice settings and multiple institutions	EAP, Germany	Eigentler, T.K. [48]	IPI	196
12, 2010	Efficacy and safety of ipilimumab monotherapy in patients with pretreated advanced melanoma: a multicenter single-arm phase II study	NCT00289627	O’Day, S.J. [54]	IPI	155
13, 2010	Ipilimumab monotherapy in patients with pretreated advanced melanoma: a randomised, double-blind, multicentre, phase 2, dose-ranging study	NCT00289640	Wolchok, J.D. [55]	IPI Three dose schedules	73/72/72

## Data Availability

The data presented in this study are available in this article.

## Appendix A. Search Strategy

### A.1. Databases

Ovid MEDLINE(R) and Epub Ahead of Print, In-Process, In-Data-Review and Other Non-Indexed Citations and Daily 1946 to 22 April 2022. EMBASE 1974 to 2022 April 22.

### A.2. MEDLINE

1. PD-1-inhibitor.ab,kf,ti.
2. exp Programmed Cell Death 1 Receptor/
3. Anti-PD-1.ab,kf,ti.
4. exp Melanoma/
5. cutaneous melanom.ab,kf,ti.
6. skin melanoma.ab,kf,ti.
7. exp Nivolumab/
8. nivolumab.ab,kf,ti.
9. opdivo.ab,kf,ti.
10. exp Ipilimumab/
11. ipilimumab.ab,kf,ti.
12. yervoy.ab,kf,ti.
13. pembrolizumab.ab,kf,ti.
14. keytruda.ab,kf,ti.
15. «adverse event*».ab,kf,ti.
16. *adverse effect*».ab,kf,ti.
17. “Drug-Related Side Effects and Adverse Reactions”/
18. (Immune adj3 toxicity).ab,kf,ti.
19. (Treatment adj toxicity).ab,kf,ti.
20. exp Hepatitis/
21. hepatitis.ab,kf,ti.
22. exp Colitis/
23. colitis.ab,kf,ti.
24. exp Pneumonia/
25. pneumonia.ab,kf,ti.
26. pneumonitis.ab,kf,ti.
27. CTLA-4-inhibitor.ab,kf,ti.
28. exp CTLA-4 Antigen/
29. Anti-CTLA-4.ab,kf,ti.
30. 1 or 2 or 3 or 7 or 8 or 8 or 10 or 11 or 12 or 13 or 14 or 27 or 28 or 29
31. 4 or 5 or 6
32. 15 or 16 or 17 or 18 or 19, or 20 or 21 or 22 or 23 or 24 or 25 or 26
33. 30 and 31 and 32
34. Programmed Cell Death Receptor 1.ab,kf,ti.
35. CTLA-4 Antigen.ab,kf,ti.
36. 30 or 34 or 35
37. 31 and 32 and 36

### A.3. EMBASE

1. exp cutaneous melanoma/
2. cutaneous melanoma.ab,kf,ti.
3. skin melanoma.ab,kf,ti.
4. exp programmed death 1 receptor/
5. Programmed Cell Death 1 Receptor.ab,kf,ti.
6. PD-1-inhibitor.ab,kf,ti.
7. Anti-PD-1.ab,kf,ti.
8. exp cytotoxic T lymphocyte antigen 4/
9. CTLA-4 Antigen.ab,kf,ti.
10. CTLA-4 inhibitor.ab,kf,ti.
11. Anti-CTLA-4.ab,kf,ti.
12. exp nivolumab/
13. Nivolumab.ab,kf,ti.
14. Opdivo.ab,kf,ti.
15. exp ipilimumab/
16. Ipilimumab.ab,kf,ti.
17. Yervoy.ab,kf,ti.
18. exp pembrolizumab/
19. Pembrolizumab.ab,kf,ti.
20. Keytruda.ab,kf,ti.
21. “adverse event*”.ab,kf,ti.
22. “adverse effect*”.ab,kf,ti.
23. adverse event/
24. (Immune adj3 toxicity).ab,kf,ti.
25. (Treatment adj toxicity).ab,kf,ti.
26. hepatitis/
27. Hepatitis.ab,kf,ti.
28. exp colitis/
29. Colitis.ab,kf,ti.
30. exp pneumonia/
31. Pneumonia.ab,kf,ti.
32. Pneumonitis.ab,kf,ti.
33. 1 or 2 or 3
34. 4 or 5 or 6 or 7 or 8 or 9 or 10 or 11 or 12 or 13 or 14 or 15 or 16 or 17 or 18 or 19 or 20
35. 21 or 22 or 23 or 24 or 25 or 26 or 27 or 28 or 29 or 30 or 31 or 32
36. 33 and 34 and 35
